# Cases of Hemorrhoids, Treated by Nitric Acid

**Published:** 1850-03

**Authors:** 


*Cases of Hemorrhoids, treated by Nitric Acid.* By Dr. R. T. Massy, Physician to the Exeter Dispensary.

Pure nitric acid performs most rapid and wonderful cures in bleeding piles, whether external or internal, and is a safe and easy remedy in its application Since Mr. Cusack, of Madam Stephen's Hospital, in Dublin, first introduced it into practice, I have frequently recommended surgeons to try it before using the ligature or knife. I can assert, from some experience in using it myself, and from the writings and remarks of others, that we have not a better or safer application to *cure* hemorrhoids.

One gentleman, who consulted me in May, 1847, describes his piles as hanging about his legs. I shall give the notes from my medical case-book of record:

---

*In these cases, a useful medicine is the phosphate of lime, ten or fifteen grains once or twice a day, mixed with the food of the child.—Ed.

A. B., aged 30, a farmer, of fair complexion, stature five feet six inches, countenance anæmic, has been an invalid for some years with bleeding piles, which came on gradually: and to them he has a family predisposition. Present state of the complaint:—Has got a large vascular tumour of the rectum protruding at the verge of the anus, which bleeds half a pint of black blood immediately after stool. The tumour has two bleeding surfaces, with an excoriation of almost an inch square. His bowels are moved at ten o'clock, P. M., every night, by taking at noon each day (which is an hour before his luncheon,) a wine-glassful of the following aperient mixture:—

℞.—Infusi sennæ, ℥vi.;

Sulph. magnesia, ʒiv.;

Carb magnesia, ℥iss.

M. ft. mist.

One night, after stool, as he lay on his side on a mattrass, I applied, with the feather of a quill, nitric acid to the bleeding surface; and when the part turned white, I laid on a pledgit of lint, covered with olive oil, and then returned the gut. Complained of slight darting pains during the night. Got up on the second day; made an examination on the twelfth day; the tumour is greatly reduced in size, and bleeds very little now; applied the nitric acid as before. Remained in bed for a day; felt a degree of tightness during defecation, in the region of the anus.

24th day.—Applied the nitric acid again to contract a small remaining portion of the tumour.

36th day.—Is now cured, can ride and drive, and walk about during the whole day; feels a new man.

I have touched with the acid a bleeding surface three inches above the rectum, with the same benefit.

A. C.—This man lost, at each stool, a large quantity of blood; he was quite emaciated and worn out with pains in the back, and down the thighs. On looking up the rectum I discovered an oval bleeding surface, its greatest diameter being more than an inch; it was soft, vascular, and elevated, and about three inches from the verge of the anus. Two applications of the acid cured the patient.

A. D., aged 27; this gentleman had external blind piles; two were visible at the verge of the anus, feeling tense, and looking as large as a hazel nut. Touched them with nitric acid. On the fourth day they were quite contracted, and drawn up. It is now more than twelve months since he was cured, and he has had no return of them since. Persons having hemorrhoids should always be advised to defecate just before going to bed; this can be easily arranged by giving a dinner pill,—say three grains of the pilulæ aloes dilute with one grain of gum mastic.

As this pill mass is not given in the Pharmacopœia, I may state its composition:—Pilulæ aloes dilutæ, *Jephson.*

℞.—Aloes bbs., saponis hispan., ext. glycyrrh., theriacæ, sing., partes æquales; solve in aqua; cola spissa leni calore.

Although I always look to the state of the liver, and give a blue pill at bed-time, and a saline aperient early on the following morning, that the bile may be removed, and the bowels cleared out before using the acid, yet I look on many cases as local, and by no means connected with the portal circulation.

I am in the habit of desiring the patient, after I have used the acid, to lie on a sofa, or to remain in bed for the day; notwithstanding this injunction, I have now and then met one, on the sofa-day, at a dinner party, or walking about town, the pain from pure nitric acid is so slight, causing little or no inconvenience.—*Medical Times, June* 30, 1849.

